# Prosocial Personality Traits Differentially Predict Egalitarianism, Generosity, and Reciprocity in Economic Games

**DOI:** 10.3389/fpsyg.2016.01137

**Published:** 2016-08-09

**Authors:** Kun Zhao, Eamonn Ferguson, Luke D. Smillie

**Affiliations:** ^1^Melbourne School of Psychological Sciences, The University of Melbourne, MelbourneVIC, Australia; ^2^School of Psychology, University of NottinghamNottingham, UK

**Keywords:** dictator game, social preferences, honesty-humility, agreeableness, politeness, compassion, big five, HEXACO

## Abstract

Recent research has highlighted the role of prosocial personality traits—agreeableness and honesty-humility—in egalitarian distributions of wealth in the dictator game. Expanding on these findings, we ran two studies to examine individual differences in two other forms of prosociality—generosity and reciprocity—with respect to two major models of personality, the Big Five and the HEXACO. Participants (combined *N* = 560) completed a series of economic games in which allocations in the dictator game were compared with those in the generosity game, a non-constant-sum wealth distribution task where proposers with fixed payoffs selected the size of their partner’s payoff (“generosity”). We further examined positive and negative reciprocity by manipulating a partner’s previous move (“reciprocity”). Results showed clear evidence of both generosity and positive reciprocity in social preferences, with allocations to a partner greater in the generosity game than in the dictator game, and greater still when a player had been previously assisted by their partner. There was also a consistent interaction with gender, whereby men were more generous when this was costless and women were more egalitarian overall. Furthermore, these distinct forms of prosociality were differentially predicted by personality traits, in line with the core features of these traits and the theoretical distinctions between them. HEXACO honesty-humility predicted dictator, but not generosity allocations, while traits capturing tendencies toward irritability and anger predicted lower generosity, but not dictator allocations. In contrast, the politeness—but not compassion—aspect of Big Five agreeableness was uniquely and broadly associated with prosociality across all games. These findings support the discriminant validity between related prosocial constructs, and have important implications for understanding the motives and mechanisms taking place within economic games.

## Introduction

One of the major themes in the literature on economic games is that humans care about and are motivated by the interests of others. These other-regarding or social preferences are the building blocks of prosocial behavior and have been incorporated into various economic models (e.g., Fehr and Schmidt, 1999; Bolton and Ockenfels, 2000; Charness and Rabin, 2002; Falk and Fischbacher, 2006). A second major theme to emerge from this literature is the substantial heterogeneity in people’s social preferences and behaviors despite being exposed to the same experimental conditions (Fischbacher et al., 2001; Camerer, 2003; Fehr and Schmidt, 2006). Measures of social value orientation, which capture motivational differences in the distribution of resources, reveal a variety of archetypes, including altruistic, prosocial, individualistic, and competitive (Murphy and Ackermann, 2014). Recent studies have also documented stable patterns of prosocial behavior correlated over time and across different games (Yamagishi et al., 2013; Peysakhovich et al., 2014).

One potential source of this heterogeneity rests in broad dispositions capturing consistent and enduring patterns in behavior and experience. Specifically, *personality traits* are “probabilistic descriptions of relatively stable patterns of emotion, motivation, cognition, and behavior, in response to classes of stimuli that have been present in human cultures over evolutionary time” (DeYoung, 2015, p. 35). A long line of research has documented how basic prosocial personality traits—known as agreeableness and honesty-humility—contribute to experimental and real-world instances of prosociality, including helping, volunteering, charitable giving, and ethical decision making (e.g., Elshaug and Metzer, 2001; Carlo et al., 2005; Penner et al., 2005; Ozer and Benet-Martínez, 2006; Graziano et al., 2007; Ashton and Lee, 2008; Aghababaei et al., 2014). It is not surprising, then, that the same prosocial traits have begun to emerge as significant predictors of inequality aversion, egalitarianism, and fairness in economic games (Hilbig et al., 2014; Zhao et al., 2016; for a review, see Zhao and Smillie, 2015).

In the current paper, we extend this nascent literature by applying a framework of distinct prosocial traits to a broader range of social preferences beyond egalitarianism. We first present an overview of the prosocial domains of major personality models and discuss their relevance for distributive and reciprocal preferences in economic games. Building on the design of the traditional dictator game, we develop a novel paradigm that simultaneously tests for two other forms of social preference beyond egalitarianism: generosity and reciprocity.

### Prosocial Domains of Major Personality Models

Prosociality is a general term referring to a variety of positive emotions, attitudes, and behaviors directed toward others, which may be manifested through acts of sharing, helping, and cooperating (Knafo-Noam et al., 2015). There is increasing recognition that neither prosociality nor its underlying motivations are unitary constructs (Batson and Powell, 2003; Singer and Steinbeis, 2009; Böckler et al., 2016). Likewise, there are multiple prosocial tendencies, which are classified differently according to two major taxonomic models of personality, the Big Five (Goldberg, 1981; Digman, 1990; John et al., 2008; DeYoung, 2015) and the HEXACO (Honesty-Humility, Emotionality, eXtraversion, Agreeableness, Conscientiousness, Openness to Experience; Lee and Ashton, 2004).

#### Prosocial Domains of the Big Five: Agreeableness and Its Aspects of Politeness and Compassion

The Five-Factor Model or “Big Five” is a robust hierarchical taxonomy of personality dimensions recovered from a number of measures of trait descriptors (John et al., 2008) and replicable across languages and cultures (Digman, 1990). Each factor represents a major dimension of covariation among traits, subsuming a number of narrower personality characteristics at intermediate (known as *aspects*) and lower (known as *facets*) levels (DeYoung et al., 2007; DeYoung, 2015).

Within the Big Five model, agreeableness captures tendencies toward altruism and cooperation, and has a core underlying motivation of maintaining interpersonal harmony (Graziano and Eisenberg, 1997). Consistent with this, agreeableness is the Big Five dimension most frequently associated with prosocial behaviors in a variety of economic games, including allocations of wealth in the dictator game (Ben-Ner et al., 2004; Becker et al., 2012; Baumert et al., 2014), acceptance of unfair offers in the ultimatum game (Mehta, 2007), cooperation in the prisoner’s dilemma (Kagel and McGee, 2014), contributions in the public goods game (Volk et al., 2011), and amounts invested and returned to others in the trust game (Evans and Revelle, 2008; Becker et al., 2012; for a review, see Zhao and Smillie, 2015).

However, Big Five agreeableness is a broad domain of personality which can be divided into two distinct aspects: *politeness*, the tendency to respect others, adhere to social norms, and suppress aggressive impulses, and *compassion*, the tendency to be emotionally concerned about others (DeYoung et al., 2007; DeYoung, 2015). Though correlated, the two often show diverging associations with other individual differences. For instance, while politeness is associated with the moral foundation of authority/respect and political conservatism, compassion is more strongly linked with the moral foundations of harm/care and fairness/reciprocity, as well as political liberalism (Hirsh et al., 2010; Osborne et al., 2013). This distinction between politeness and compassion also has important implications for the study of heterogeneity in economic games, where prosocial behaviors in different games may stem from different motivations, such as adhering to normative rules around sharing and cooperating (e.g., the public goods game), or helping needy others (e.g., third party punishment and recompensation).

#### Prosocial Domains of the HEXACO: Honesty-Humility and Agreeableness

A major alternative to the Big Five is the HEXACO model, a six-factor model of personality developed from psycholexical studies in European and Asian languages (Lee and Ashton, 2004; Ashton and Lee, 2007; Ashton et al., 2014). The most salient difference between the HEXACO and the Big Five is the addition of a sixth dimension, honesty-humility, or the tendency to be sincere, modest, and fair, which is believed to capture trait variance beyond the Big Five. Moreover, the HEXACO representation of agreeableness and emotionality (neuroticism) are rotational variants of their Big Five counterparts (Ashton and Lee, 2007). Specifically, HEXACO agreeableness reflects the tendency to be patient, forgiving, and tolerant, and is thus non-interchangeable with Big Five agreeableness, which reflects broad tendencies toward altruism.

Together, HEXACO honesty-humility and HEXACO agreeableness span the prosocial domain typically captured by Big Five agreeableness and make up two forms of individual variation in reciprocal altruism. Honesty-Humility represents *active cooperation*, the tendency to cooperate with others despite the opportunity for exploitation, while HEXACO agreeableness represents *reactive cooperation*, the tendency to cooperate with others despite their misgivings (Hilbig et al., 2013; Ashton et al., 2014). The two diverge in studies of workplace delinquency (Lee et al., 2005), criminality (Rolison et al., 2013), dishonesty and cheating (Hilbig and Zettler, 2015), and forgiveness and revenge (Lee and Ashton, 2012). This discriminant validity is also relevant to behavior within economic games, where there is evidence of a “cooperative phenotype,” characterized by within-individual correlations across cooperative games (i.e., fair and cooperative tendencies corresponding to honesty-humility), which is independent from norm-enforcing punishment (i.e., retaliatory tendencies corresponding to HEXACO agreeableness; Peysakhovich et al., 2014).

In summary, the Big Five and HEXACO models provide an array of distinct prosocial traits which reflect different motivations and mechanisms, and which show divergent validity with respect to interpersonal and socio-political variables (see **Table 1**). We now turn to the experimental economics literature, where similar distinctions may exist between different facets of prosociality and which are expressed through multiple social preferences in games.

**Table 1:** Prosocial domains of the Big Five and HEXACO models of personality.

Personality dimension	Defining characteristic	Known roles in relevant games
**Big Five model**		
Agreeableness	Broad tendencies toward altruism and cooperation	• Fair allocations of wealth • Amount returned in trust game • Acceptance in ultimatum game
Politeness	Tendency to adhere to social norms; alignment with the group	• Fair allocations of wealth
Compassion	Tendency to be emotionally concerned about others; alignment with another individual	
**HEXACO model**		
Honesty-Humility	Tendency to cooperate despite opportunities for exploitation; active cooperation	• Fair allocations of wealth • Amount returned in trust game
Agreeableness	Tendency to cooperate despite the misgivings of others; reactive cooperation	• Acceptance in ultimatum game

*For a discussion of the role of empathic concern (compassion) as alignment with other individuals and social norms (politeness) as alignment to one’s group, see Jensen et al. (2014). Within the HEXACO model, honesty-humility and agreeableness are thought to represent two complementary aspects of reciprocal altruism. In addition, HEXACO emotionality, the tendency to be sentimental and oversensitive, is believed to relate to the construct of kin altruism (Ashton and Lee, 2007; Ashton et al., 2014). However, this dimension is beyond the scope of the current research, which focuses on prosocial behavior among non-kin.*

### Multiple Social Preferences in Economic Games

#### Inequality Aversion and Egalitarianism

One basic way in which social preferences deviate from narrow self-interest is the desire for equality. Egalitarianism is a basic motivation that can be traced back to small-scale societies in human evolutionary history (Boehm, 1999) and is the cornerstone of economic theories of social preferences (Loewenstein et al., 1989; Fehr and Schmidt, 1999; Bolton and Ockenfels, 2000). The tension between self-interest and equality is best captured in the dictator game, in which one player decides how to split a fixed amount of money with a second player, who must accept this unconditionally (Kahneman et al., 1986; Forsythe et al., 1994). Featuring in more than a hundred studies, the popularity of the dictator game owes to the fact that it is a simple yet powerful paradigm which yields considerable behavioral variation (Engel, 2011). While average allocations to a partner range between 20% and 30% of the pie, up to half of participants keep all the money, a quarter split it equally, and the remainder select distributions in between (Tisserand et al., 2015). This heterogeneity thus makes the dictator game an ideal hunting ground for examining the influence of personality and for teasing apart the roles of similar but distinct personality constructs.

For example, Big Five agreeableness is a consistent predictor of egalitarian dictator allocations (for a review, see Zhao and Smillie, 2015). However, recent research indicates that this is driven by its aspect of politeness—or tendencies toward good manners and etiquette—rather than compassion (Zhao et al., 2016), in keeping with the economics literature on the importance of social norms for prosociality (Camerer and Thaler, 1995). Another kind of dissociation has emerged within the HEXACO model, with several studies showing that honesty-humility (or the tendency for active cooperation)—but not HEXACO agreeableness—is a strong, consistent, and robust predictor of egalitarian dictator allocations, and even more so than Big Five agreeableness (Hilbig and Zettler, 2009; Thielmann and Hilbig, 2014; Hilbig et al., 2015a, 2013; Zhao et al., 2016; for a review, see Zhao and Smillie, 2015).

#### Costless Prosociality and Generosity

Despite the wealth of findings it has generated, the dictator game is limited when drawing inferences about a wider array of social preferences. Notably, the constant-sum structure of the game means that decisions to benefit one’s partner are always at a cost to self-interest by the same magnitude. However, many instances of real-world prosociality involve decisions which benefit others at minimal personal cost, such as giving pre-loved belongings to charity and posthumous organ donation (Saunders, 2012; Moorlock et al., 2014; Shepherd et al., 2014). In this paper, we use the term *generosity* to describe the willingness to accept a relative disadvantage when this makes others better off (either at a personal cost or at no cost), but it should not be confused with other usages in the literature (e.g., Haley and Fessler, 2005).

Acts of generosity are typically obscured by dominant norms of equality in constant-sum games, such as the dictator game, where fewer than 5% of individuals allocate more than half the endowment to their partner (Tisserand et al., 2015). However, acts of generosity emerge in tasks of costless prosociality where they may reflect concerns for efficiency and social welfare (Charness and Rabin, 2002; Fehr et al., 2008; Bartling et al., 2009; Güth, 2010). In their study of egalitarianism in children, Fehr et al. (2008) used an envy game in which participants chose between one unit each (1,1) or one for themselves and two for their partner (1,2), finding that although egalitarian preferences dominated at ages 7–8, they were gradually replaced by generosity in older ages (Fehr et al., 2013).

In adults, costless prosociality has been incorporated into modified dictator games consisting of simple allocation tasks, such as selecting an efficient but personally disadvantageous (400,750) choice over an egalitarian (400,400) one (Charness and Rabin, 2002; Engelmann and Strobel, 2004). The generosity game has been specifically designed to examine efficiency concerns, in which individuals choose the size of the overall pie when their own share is fixed (Güth, 2010; Güth et al., 2012). When there is no trade-off between self- and other-interests, most individuals maximize their partner’s payoff, with a substantial portion preferring equal shares and a minority minimizing their partner’s payoffs (Güth et al., 2012). At the other end of the spectrum, choosing to hurt another or refusing to help them when there is little personal gain may represent purer forms of spite or envy (Abbink and Sadrieh, 2009). Studies using joy-of-destruction games show that some individuals—almost 40% of concealed game decisions—are willing to reduce the payoffs of others even when they do not benefit directly (Abbink and Sadrieh, 2009; Zhang and Ortmann, 2016). Clearly there is much individual variation in costless prosocial and antisocial behaviors—perhaps more so than when decisions are costly and self-interest is a strong driver of uniform responding—and these differences may be reconciled by examining the role of relevant personality constructs, including tendencies toward benevolence, lenience, and spite.

#### Positive and Negative Reciprocity

In addition to distributive preferences that govern egalitarianism and generosity, another major influence deeply embedded within social interactions are reciprocal preferences (Fehr and Gächter, 2000; Charness and Rabin, 2002; Dufwenberg and Kirchsteiger, 2004; Falk and Fischbacher, 2006). Reciprocity is the tendency to return others’ favors and to retaliate against others’ wrongdoing (Gouldner, 1960) and is believed to underlie the evolution and maintenance of human cooperation (Axelrod and Hamilton, 1981; Komorita and Parks, 1999; Bowles and Gintis, 2004). In economics, behavioral signatures of positive and negative reciprocity are often studied in the second player roles of the trust and ultimatum games, respectively (e.g., Fehr et al., 2002; Becker et al., 2012).

Individual differences in the tendency to reciprocate are well documented (Gallucci and Perugini, 2000; Ackermann et al., 2014), and self-reported reciprocity is associated with major life and economic outcomes (Dohmen et al., 2009). However, the exact relations between positive and negative reciprocity and narrower personality traits are less clear, particularly given the highly conditional nature of reciprocity. For example, positive reciprocators not only need to be sensitive to positive gestures from others, but also have a behavioral propensity to respond to these positively (Perugini et al., 2003).

Within the Big Five model, self-reported positive reciprocity is positively correlated with agreeableness and conscientiousness, while negative reciprocity is negatively correlated with the same two traits, and positively with neuroticism (Perugini et al., 2003; Dohmen et al., 2008). Interestingly, all three traits predict the same outcomes—work effort, unemployment, and subjective wellbeing—associated with individual differences in negative and positive reciprocity, providing further evidence of their overlap (Ozer and Benet-Martínez, 2006; Dohmen et al., 2009). Consistent with these self-reported findings, agreeableness is the Big Five trait most frequently associated with reciprocal behavior in economic games, where it predicts the acceptance of unfair offers in the ultimatum game (Mehta, 2007; Li and Chen, 2012) and greater amounts returned to a sender in the trust game (Evans and Revelle, 2008; Ben-Ner and Halldorsson, 2010; Becker et al., 2012; Müller and Schwieren, 2012; but see Thielmann and Hilbig, 2015).

Furthermore, the HEXACO model and its partitioning of the prosocial domain into active (i.e., honesty-humility) and reactive (i.e., agreeableness) forms of reciprocal altruism is ideally suited to the finer-grained analysis of positive and negative reciprocity in economic games. HEXACO agreeableness has been negatively associated with self-reported negative reciprocity (Perugini et al., 2003) and shown to predict acceptance of unfair offers in ultimatum games (Hilbig et al., 2013; Thielmann et al., 2014). Meanwhile, honesty-humility has been found to predict trustworthiness, measured by the amount returned in the trust game (Thielmann and Hilbig, 2015). However, this was independent of prior trust, suggesting that the relation is likely driven by a mechanism of “unconditional kindness” (i.e., giving in the absence of any previous or future interaction with one’s partner, such as in a one-shot dictator game), rather than positive reciprocity *per se* (Thielmann and Hilbig, 2015). Other research using wealth redistribution paradigms similarly found that the behavioral expression of honesty-humility is less conditional on fairness norms overall and instead resembles an overall pattern of benevolence (Hilbig et al., 2015b).

### The Current Research

Social preferences represent a number of channels through which humans deviate from narrow self-interest and engage in prosocial behaviors. Distributive preferences capture concerns for egalitarianism and generosity, while preferences for reciprocity promote favorable or unfavorable treatment conditional on the previous acts or intentions of others. Emerging research has demonstrated considerable heterogeneity in these preferences, which may be partially underpinned by prosocial personality traits. However, most of this research has focused on the trade-off between self- and other-regarding interests in the dictator game. Detailed relations between prosocial personality traits and other forms of social preferences are less well understood, and inferences are often cobbled together from a mixture of different games and personality measures. As a result, it is difficult to disentangle trait effects from the influence of contextual factors across variable game environments (given that traits too are contextualized; DeYoung, 2015) and to interpret the findings when certain game decisions are used to approximate social preferences (e.g., the trust game, which may not capture positive reciprocity; Ben-Ner and Halldorsson, 2010; Thielmann and Hilbig, 2015).

The aims of the current research were threefold: (1) To identify a richer set of social preferences beyond egalitarianism and inequality aversion, (2) to examine the source of individual differences in these preferences using theoretical models of distinct prosocial traits, and in doing so, (3) address some of the major limitations of the existing literature (e.g., fragmented games and traits).

We developed a novel paradigm using six simple modifications of the dictator game to test multiple social preferences. This design was inspired by Charness and Rabin (2002), who incorporated reciprocity and efficiency concerns into a series of binary-choice tasks. We first manipulated the costliness of decisions by setting half the games as constant-sum (i.e., costly dictator games) and half with a fixed personal payoff but variable partner payoff (i.e., costless generosity games). Second, we manipulated the conditions for reciprocity by positioning these games after a prior decision by a partner that hurt or helped the participant, vs. a baseline condition where there was no history with a partner.

The benefit of this design is that it provided a suite of tightly controlled and manipulable conditions ideal for localizing specific prosocial constructs. For example, comparing costly vs. costless game decisions allowed us to identify different patterns of behavior after controlling for the influence of self-interest. Similarly, reciprocal tendencies can be teased apart from overall altruistic motivations. Existing studies suggest that Big Five agreeableness and HEXACO honesty-humility are associated positively with positive reciprocity and negatively with negative reciprocity, but these preferences have been largely considered in isolation. Given that these traits are already associated with greater dictator allocations, the current design will reveal whether they produce an *additional* effect for reciprocity, above and beyond unconditional kindness (Ben-Ner and Halldorsson, 2010; Thielmann and Hilbig, 2015).

We then examined the sources of heterogeneity within this paradigm with respect to the theoretically relevant prosocial domain of personality: agreeableness and its aspects of politeness and compassion within the Big Five model, and honesty-humility and agreeableness within the HEXACO model. In particular, we focused on the discriminant validity between similar prosocial personality constructs and identified unique trait effects to help shed light on the specific mechanisms and motivations taking place within economic games (for a recent example, see Zhao et al., 2016). We sought to address some of the limitations and expand on the existing research by bringing together two large and relatively diverse community samples. Sample sizes in both studies (*N*s = 304, 256) were well above the recommended minimum provided in the wake of the replicability crisis, including total *N* > 150 for individual differences research (Mar et al., 2013) and total N > 180 as a general requirement for personality and social psychological research (Vazire, 2016). As the design was within-subjects, the *per condition* sample sizes provided 80% power to identify effect sizes of approximately *r*s = 0.16–0.18 (Faul et al., 2009), which is reasonably sensitive given the average effect sizes in the field (*r* = 0.21; Richard et al., 2003; Fraley and Marks, 2007).

In line with previous research, we expected politeness from the Big Five model to be uniquely associated with costly prosocial allocations (i.e., dictator games) but expected compassion to play a relatively stronger role in costless prosocial allocations (i.e., generosity games), where allocations are less norm-driven and capture motivations of improving the wellbeing of others. Within the HEXACO model, we predicted that honesty-humility would have a unique role in both costly and costless prosociality, given its core characteristic of benevolence. Furthermore, we hypothesized that HEXACO agreeableness—which captures tendencies toward forgiveness and non-retaliation—would be negatively associated with negative reciprocity. Finally, in light of the evidence demonstrating the role of Big Five agreeableness and HEXACO honesty-humility in *both* positive reciprocal game behaviors and dictator game allocations, we were interested in examining whether any prosocial traits could explain positive reciprocity beyond their established role in unconditional kindness.

## Study 1

### Materials and Methods

#### Ethics Statement

This study was approved by the Human Ethics Advisory Group of the Melbourne School of Psychological Sciences, The University of Melbourne. All participants provided informed consent via an electronic survey according to the established guidelines of the Group.

#### Participants

The final sample consisted of 304 North American participants (aged 18–65 years, *M*_age_ = 30.90, *SD* = 9.89; 55% female) recruited from Amazon Mechanical Turk (MTurk). Only workers with fewer than 50 Human Intelligence Tasks were selected to avoid recruiting those who were familiar with economic game paradigms.

#### Personality Measures

##### Big Five Aspect Scales (BFAS; DeYoung et al., 2007)

Participants completed the 100-item BFAS, a measure of the five broad domains of personality (neuroticism, agreeableness, conscientiousness, extraversion, and openness/intellect) and their lower-level aspects. Of particular interest was the prosocial domain of agreeableness, including its aspects of politeness (e.g., “insult people”) and compassion (e.g., “inquire about others’ wellbeing”). These were each measured with 10 items on a five-point Likert scale (1 = strongly disagree, 5 = strongly agree). The BFAS is a well-validated measure of the Big Five and has good internal consistency and test–retest reliability (DeYoung et al., 2007).

##### HEXACO Personality Inventory—Revised (HEXACO-PI-R; Lee and Ashton, 2004)

Participants also completed the 100-item HEXACO-PI-R, an alternative measure of personality comprising six broad trait domains. Of particular interest were the prosocial domains of honesty-humility (e.g., “I am an ordinary person who is no better than others”) and agreeableness (e.g., “I rarely hold a grudge, even against people who have badly wronged me”). Each trait is measured with 16 items on a five-point Likert scale (1 = strongly disagree, 5 = strongly agree), and has good internal consistency (Lee and Ashton, 2004)^1^.

#### Procedure

Participants completed demographic questions, personality measures, and economic games on a survey programmed using Qualtrics Survey Software and administered through the MTurk requester interface. The BFAS and the HEXACO-PI-R were presented one after the other in a randomized order. The survey consisted of additional questionnaires and economic games beyond the scope of the current research, including a hypothetical real-world economic decision-making task. The 200 items of the personality questionnaires served as a filler task between this and the current games of interest, and thus were expected to prevent any carryover effects.

All economic games were hypothetical, that is, participants were asked to imagine that they were playing the games with an anonymous partner who was described as another participant that they would not knowingly meet. To check the validity of responses, participants also completed two attention checks embedded in the personality measures (e.g., “Please select Strongly Agree”). Thirty-six (11%) participants were excluded for failing at least one of these attention checks. Participants were paid US$2.00 and the median time spent on the study was 30 min.

##### Economic games

Participants played six economic games that were loosely based on a larger set of dictator and response games developed by Charness and Rabin (2002). All six games required the participant to select their preferred choice out of 11 combinations of payoffs for themselves and their partner, represented by imagined dollar amounts. All games were presented in a randomized order.

**FIGURE 1:**
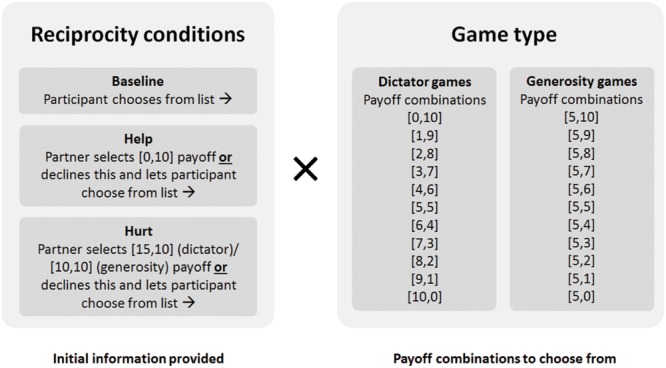
**Study design depicting the three reciprocity conditions and the two game types.** Payoffs were represented as imagined dollars (Study 1) or points corresponding to dollar amounts (Study 2). Participants’ payoffs are always listed first in payoff combinations.

The six games were set up using a 2 (game type: dictator vs. generosity) × 3 (reciprocity: baseline, help, and hurt) repeated measures design, depicted in **Figure 1.** There were two types of games: dictator and generosity games. In the three dictator games (Kahneman et al., 1986; Forsythe et al., 1994), participants were asked to indicate their preferred choice out of 11 different payoff combinations, each of which summed to $10. These ranged from $0 for oneself and $10 for one’s partner to $10 for oneself and $0 for one’s partner, varying in $1 increments.

In the three generosity games (based on Güth et al., 2009, 2012; Güth, 2010)^2^, participants were again asked to indicate their preferred selection out of 11 different payoff combinations. This time, their own payoff was always fixed at $5 and the choices ranged from $0 to $10 for their partner, varying in $1 increments.

In addition, there were three types of reciprocity conditions: baseline, help, and hurt. In the two baseline games, participants were asked to indicate their preferred selection with no information provided about their partner. In the four remaining games, participants were provided information about their partner’s previous move, which involved passing on a decision that either helped or hurt the participant. In the two help games, participants read that their partner had passed on a decision with a payoff of $0 to the participant, opting instead to defer to the participant to choose from the list of current options. In the two hurt games, participants read that their partner had passed on a decision with a payoff of $15 (dictator version) or $10 (generosity version) to the participant, opting instead to defer to the participant. In other words, the partner’s move in the help condition prevented the participant from going away empty-handed, while their move in the hurt condition resulted in the participant missing out on $15 (dictator version) or $10 (generosity version). These different forgone payoffs between the dictator and generosity games correspond to the maximum amounts that could be earned in each of these games ($10 in the dictator game, $5 in the generosity game).

To summarize, this experimental setup would thus reveal an effect for generosity if there were greater allocations in the generosity games relative to the dictator game (i.e., a main effect for game type). In addition, reciprocity would be evident from varying allocations of wealth between the baseline, help, and hurt games (i.e., a main effect of reciprocity), in which higher allocations in the help games would be indicative of positive reciprocity and lower allocations in the hurt games indicative of negative reciprocity.

### Results and Discussion

#### Preliminary Statistics

##### Game decisions

**FIGURE 2:**
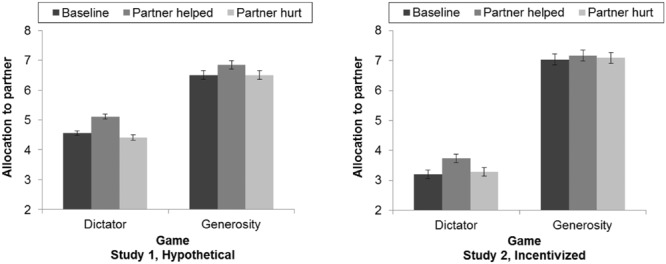
**Mean allocations to partner across all games.** Error bars represent one standard error. *N* = 304 (Study 1), 256 (Study 2).

Mean allocations to a partner in each of the six economic bargaining games are presented in the left panel of **Figure 2.** A 2 (game type: dictator vs. generosity) × 3 (reciprocity: baseline, help, and hurt) repeated measures ANOVA was performed. Greenhouse-Geisser corrections were applied for sphericity violations of reciprocity, χ^2^(2) = 29.92, *p* < 0.001 (𝜀 = 0.91), and its interaction with game type, χ^2^(2) = 14.62, *p* = 0.001 (𝜀 = 0.96). There was a main effect for game type, with allocations in generosity games (*M* = 6.62) higher than those in dictator games (*M* = 4.70), *F*(1,303) = 212.12, *p* < 0.001, η_p_^2^ = 0.41. There was also a main effect for reciprocity, *F*(1.83,553.77) = 15.68, *p* < 0.001, η_p_^2^ = 0.05, for which allocations in the baseline games (*M* = 5.54) were significantly lower than in help games (*M* = 5.98), *F*(1,303) = 27.16, *p* < 0.001, η_p_^2^ = 0.08, but not in hurt games (*M* = 5.46), *F*(1,303) = 0.57, *p* = 0.45, η_p_^2^ = 0.002. These findings thus indicate generosity and positive reciprocity, but not negative reciprocity.

##### Demographic variables

Age and gender are important demographic variables frequently associated with social preferences (Andreoni and Vesterlund, 2001; for a discussion of age-related effects and possible confounds, see Kettner and Waichman, 2016). In the current study, age was not significantly correlated with any game decisions. In contrast, there was a significant interaction between gender and game type. After removing three participants who identified as neither male nor female, gender was included in the 2 (game type) × 3 (reciprocity) repeated measures ANOVA. This model produced a main effect for gender, *F*(1,299) = 7.45, *p* = 0.01, η_p_^2^ = 0.02, with men allocating on average more than women, *M*s = 5.86 vs. 5.50, *t*(299) = 2.73, *p* = 0.01. However, these findings were moderated by a significant interaction between gender and game type, *F*(1,299) = 10.88, *p* = 0.001, η_p_^2^ = 0.04, and between gender and reciprocity, *F*(1.84,550.31) = 5.59, *p* = 0.01, η_p_^2^ = 0.02.

**FIGURE 3:**
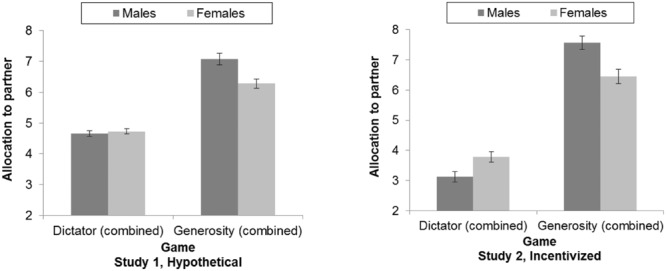
**Mean allocations to partner by game type and gender.** Data are collapsed across reciprocity conditions. Error bars represent one standard error. *N* = 301 (Study 1), 256 (Study 2).

Allocations by gender and game type are presented in the left panel of **Figure 3**, collapsed across reciprocity conditions. The main effect of gender appeared to be driven by men allocating more than women in generosity games, *M*s = 7.07 vs. 6.28, *t*(299) = 3.41, *p* = 0.001, but no differently in dictator games, *t*(299) = -0.58, *p* = 0.56. Meanwhile, men allocated more than women in the baseline, *M*s = 5.76 vs. 5.37, *t*(299) = 2.30, *p* = 0.02, and help conditions, *M*s = 6.36 vs. 5.68, *t*(299) = 4.00, *p* < 0.001, but not in the hurt conditions, *M*s = 5.47 vs. 5.46, *t*(299) = 0.05, *p* = 0.96. All main effects for game type and reciprocity were replicated when including gender.

##### Prosocial personality traits

Bivariate correlations between prosocial personality traits are shown in **Table 2** and were generally consistent with previous research (Barford et al., 2015; Zhao et al., 2016). Both HEXACO honesty-humility and, to a lesser extent, HEXACO agreeableness were more strongly correlated with politeness (*r*_s_s = 0.51, 0.26) than compassion (*r*_s_s = 0.24, 0.14).

**Table 2:** Correlations between prosocial personality traits.

		Study 1: Hypothetical					Study 2: Incentivized				
		1	2	3	4	5	1	2	3	4	5
(1)	B5 Agreeableness	0.86					0.91				
(2)	B5 Politeness	0.84^∗∗^	0.74				0.85^∗∗^	0.82			
(3)	B5 Compassion	0.84^∗∗^	0.44^∗∗^	0.88			0.90^∗∗^	0.56^∗∗^	0.92		
(4)	HEX Honesty-Humility	0.44^∗∗^	0.51^∗∗^	0.24^∗∗^	0.82		0.41^∗∗^	0.48^∗∗^	0.27^∗∗^	0.87	
(5)	HEX Agreeableness	0.22^∗∗^	0.26^∗∗^	0.14^∗^	0.19^∗∗^	0.84	0.53^∗∗^	0.52^∗∗^	0.42^∗∗^	0.31^∗∗^	0.88

*Correlations calculated using Spearman’s rho. Cronbach’s αs are shown in the diagonal. B5, Big Five model, measured using the Big Five Aspect Scales (BFAS; DeYoung et al., 2007). HEX, HEXACO model, measured using the HEXACO Personality Inventory—Revised (HEXACO-PI-R; Lee and Ashton, 2004). *N* = 304 (Study 1), 256 (Study 2). ^∗^*p* < 0.05, ^∗∗^*p* < 0.01.*

#### Personality Predictors of Game Allocations

##### Bivariate correlations

Bivariate correlations between game allocations and prosocial personality traits are shown in **Table 3** (see Supplementary Tables S1 and S2 in the Supplementary Material for correlations with all personality traits). The politeness and compassion aspects of Big Five agreeableness were both correlated with all three dictator games (*r*_s_s = 0.13–0.19). Similarly, HEXACO honesty-humility was associated with dictator (*r*_s_s = 0.12–0.26), but not generosity (*r*_s_s = -0.06 to -0.01) allocations. In contrast, HEXACO agreeableness was correlated with generosity (*r*_s_s = 0.15–0.18) but not dictator allocations (*r*_s_s = -0.01–0.10).

**Table 3:** Correlations between prosocial personality traits and game allocations.

	Study 1: Hypothetical						Study 2: Incentivized					
	DG	DG_0_	DG_15_	GG	GG_0_	GG_10_	DG	DG_0_	DG_15_	GG	GG_0_	GG_10_
**Big Five model**												
Agreeableness	0.20^∗∗^	0.17^∗∗^	0.16^∗∗^	0.02	0.05	0.01	0.19^∗∗^	0.11	0.18^∗∗^	-0.01	0.0003	-0.07
Politeness	0.17^∗∗^	0.16^∗∗^	0.15^∗∗^	0.02	0.07	-0.01	0.17^∗∗^	0.12	0.15^∗^	0.07	0.07	0.04
Compassion	0.19^∗∗^	0.16^∗∗^	0.13^∗^	0.05	0.05	0.05	0.17^∗∗^	0.07	0.17^∗∗^	-0.09	-0.07	-0.14^∗^
**HEXACO model**												
Honesty-Humility	0.20^∗∗^	0.12^∗^	0.26^∗∗^	-0.06	-0.04	-0.01	0.30^∗∗^	0.21^∗∗^	0.31^∗∗^	-0.04	-0.02	-0.08
Agreeableness	0.10	0.03	-0.01	0.17^∗∗^	0.18^∗∗^	0.15^∗∗^	0.07	-0.02	0.02	0.04	0.004	-0.02

*Correlations calculated using Spearman’s rho. Game allocations indicate amount allocated to partner out of 10 units (i.e., dollars or points). Big Five traits are measured using the Big Five Aspect Scales (BFAS; DeYoung et al., 2007). HEXACO traits are measured using the HEXACO Personality Inventory—Revised (HEXACO-PI-R; Lee and Ashton, 2004). DG, baseline dictator game. DG_0_, dictator game after partner’s decision cost the participant the 0 unit payoff. DG_15_, dictator game after partner’s decision cost the participant the 15 unit payoff. GG, baseline generosity game. GG_0_, generosity game after partner’s decision cost the participant the 0 unit payoff. GG_10_, generosity game after partner’s decision cost the participant the 10 unit payoff. *N* = 304 (Study 1), 256 (Study 2). ^∗^*p* < 0.05, ^∗∗^*p* < 0.01.*

##### Repeated measures ANCOVAs

A series of 2 (game type) × 3 (reciprocity) repeated measures ANCOVAs was performed for each personality model with the relevant prosocial traits standardized and entered simultaneously as covariates. Interactions between prosocial personality traits and game type or reciprocity are presented in **Tables 4** and **5**.

**Table 4:** ANCOVA results for interactions between prosocial traits and game type.

	Study 1: Hypothetical				Study 2: Incentivized			
Interaction term	df	*F*	*p*	η_p_^2^	df	*F*	*p*	η_p_^2^
**Big Five model (B5A only)**								
Game × B5A	1, 302	0.84	0.36	0.003	1, 254	2.24	0.14	0.01
**Big Five model**								
Game × B5Pol	1, 301	0.30	0.58	0.001	1, 253	1.97	0.16	0.01
Game × B5Comp	1, 301	0.18	0.67	0.001	1, 253	7.70	0.01	0.03
**HEXACO model**								
Game × HEXH	1, 301	12.84	<0.001	0.04	1, 253	11.48	0.001	0.04
Game × HEXA	1, 301	9.62	0.002	0.03	1, 253	2.62	0.11	0.01

*B5, Big Five model, measured using the Big Five Aspect Scales (BFAS; DeYoung et al., 2007). B5A, B5 Agreeableness. B5Comp, B5 Compassion. B5Pol, B5 Politeness. HEX, HEXACO model, measured using the HEXACO Personality Inventory—Revised (HEXACO-PI-R; Lee and Ashton, 2004). HEXA, HEX Agreeableness. HEXH, HEXACO Honesty-Humility. *N* = 304 (Study 1), 256 (Study 2).*

**Table 5:** ANCOVA results for interactions between prosocial traits and reciprocity.

	Study 1: Hypothetical				Study 2: Incentivized			
Interaction term	df	*F*	*p*	η_p_^2^	df	*F*	*p*	η_p_^2^
**Big Five model (B5A only)**								
Reciprocity × B5A	1.83, 551.89	0.04	0.95	<0.001	1.89, 478.90	0.19	0.82	0.001
**Big Five model**								
Reciprocity × B5Pol	1.83, 550.24	0.22	0.78	0.001	1.89, 477.05	0.18	0.83	0.001
Reciprocity × B5Comp	1.83, 550.24	0.20	0.80	0.001	1.89, 477.05	0.002	0.99	<0.001
**HEXACO model**								
Reciprocity × HEXH	1.84, 553.30	4.42	0.02	0.01	1.89, 478.16	0.17	0.84	0.001
Reciprocity × HEXA	1.84, 553.30	0.78	0.45	0.003	1.89, 478.16	1.87	0.16	0.01

*B5, Big Five model, measured using the Big Five Aspect Scales (BFAS; DeYoung et al., 2007). B5A, B5 Agreeableness. B5Comp, B5 Compassion. B5Pol, B5 Politeness. HEX, HEXACO model, measured using the HEXACO Personality Inventory—Revised (HEXACO-PI-R; Lee and Ashton, 2004). HEXA, HEX Agreeableness. HEXH, HEXACO Honesty-Humility. *N* = 304 (Study 1), 256 (Study 2).*

Within the Big Five model, there was a main effect for agreeableness, *F*(1,302) = 6.23, *p* = 0.01, η_p_^2^ = 0.02. Replacing this with covariates for politeness and compassion initially revealed no significant main effects for either. However, as men allocated more than women overall (primarily driven by generosity game allocations) and men were significantly lower on politeness and compassion than women, we also included gender in the same model. Here, a main effect for politeness, *F*(1,297) = 5.65, *p* = 0.02, η_p_^2^ = 0.02 [and a marginally significant effect for compassion, *F*(1,297) = 4.03, *p* = 0.05, η_p_^2^ = 0.01] emerged, suggesting that politeness was related to greater allocations across all conditions (see Supplementary Tables S3–S5 in the Supplementary Material). None of the prosocial personality traits in the Big Five model interacted with game type or reciprocity.

Within the HEXACO model, there was a main effect for agreeableness, *F*(1,301) = 9.28, *p* = 0.003, η_p_^2^ = 0.03, but not honesty-humility *F*(1,301) = 0.71, *p* = 0.40, η_p_^2^ = 0.002. This was accompanied by significant interactions between honesty-humility and game type, *F*(1,301) = 12.84, *p* < 0.001, η_p_^2^ = 0.04, and agreeableness and game type, *F*(1,301) = 9.62, *p* = 0.002, η_p_^2^ = 0.03. This pattern of findings was replicated when gender was included in the model (see Supplementary Tables S3–S5 in the Supplementary Material).

**FIGURE 4:**
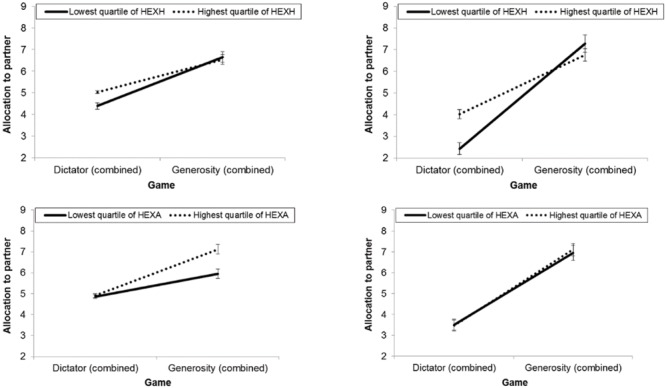
**Comparison of game allocations between the highest and lowest quartiles of HEXACO Honesty-Humility and HEXACO Agreeableness. (Left)** Study 1 (hypothetical), **(right)** Study 2 (incentivized). Data are collapsed across reciprocity conditions. Error bars represent one standard error. *N* = 304 (Study 1), 256 (Study 2).

To follow up on these interactions, we examined the effect of these two traits for dictator and generosity games separately, which revealed a “double dissociation” between the two, depicted in the left panel of **Figure 4.** Honesty-humility was uniquely associated with greater allocations in dictator games, *F*(1,301) = 23.77, *p* < 0.001, η_p_^2^ = 0.07, but did not have a main effect in generosity games, *F*(1,301) = 2.32, *p* = 0.13, η_p_^2^ = 0.01. In contrast, HEXACO agreeableness was uniquely associated with greater allocations in generosity games, *F*(1,301) = 11.88, *p* = 0.001, η_p_^2^ = 0.04, but did not have a main effect in dictator games, *F*(1,301) = 0.0004, *p* = 0.98, η_p_^2^ < 0.001.

In addition, there was a significant interaction between honesty-humility and reciprocity, *F*(1.84,553.30) = 4.42, *p* = 0.02, η_p_^2^ = 0.01. This revealed a significant main effect of honesty-humility in the hurt conditions, *F*(1,301) = 5.94, *p* = 0.02, η_p_^2^ = 0.02, but not in the baseline or help conditions (*p*s = 0.99, 0.49, respectively).

### Summary

The results of Study 1 showed clear evidence of social preferences beyond inequality aversion and egalitarianism. Individuals allocated significantly more wealth to their partners when decisions were costless than when they were costly, demonstrating tendencies toward generosity. In addition, there was evidence of positive reciprocity, with individuals allocating more wealth to their partner after their partner had assisted them. However, we found no evidence of negative reciprocity, and individuals did not allocate any differently when they had been denied a higher payoff by a hurtful partner. These findings were further moderated by gender, with men allocating more than women in the generosity games and when their partner had not previously hurt them.

The results presented a mixed picture of predicted and unexpected findings regarding the role of personality, revealing a main effect for politeness (but not so much compassion) in the Big Five model. In the HEXACO model, honesty-humility predicted greater allocations in the dictator game, in keeping with a large body of previous research (Hilbig and Zettler, 2009; Hilbig et al., 2015a; Zhao and Smillie, 2015). However, contrary to its putative mechanism of benevolence, honesty-humility did not play any role in the generosity game, where decisions were costless. Here, it was HEXACO agreeableness—or the tendency to be tolerant, lenient, and forgiving—which instead predicted greater generosity.

An important consideration in Study 1 is that the decisions were hypothetical, featuring imagined partners and stakes. Previous studies have been conflicted as to whether hypothetical paradigms produce comparable results to incentivized games, especially when trait effects are involved (Ben-Ner et al., 2008; Engel, 2011; Lönnqvist et al., 2011; Ferguson and Starmer, 2013; Hilbig et al., 2015a; Zhao et al., 2016). Another potential limitation stems from correlating self-reported personality traits with self-reported hypothetical responses, where there is a risk of inflated associations arising from common method variance (Podsakoff et al., 2003). Finally, the dearth of actual assessment of behavior has been a prominent issue in personality research, leading to calls for a broader range of data beyond self-reports and hypothetical scenarios (Funder, 2001; Baumeister et al., 2007). In light of these concerns, we ran a second study using an identical—but incentivized—paradigm with the aim of replicating our previous findings and identifying robust effects.

## Study 2

### Materials and Methods

#### Ethics Statement

This study was approved by the Human Ethics Advisory Group of the Melbourne School of Psychological Sciences, The University of Melbourne. All participants provided informed consent via an electronic survey according to the established guidelines of the Group.

#### Participants

The final sample consisted of 256 North American participants (aged 19–67 years, *M*_age_ = 34.76, *SD* = 11.00; 43% female) recruited from Amazon MTurk.

#### Personality Measures

Participants completed the 100-item BFAS (DeYoung et al., 2007), along with the honesty-humility, agreeableness, and altruism scales (see Footnote 1) from the HEXACO-PI-R (Lee and Ashton, 2004), described in Study 1.

#### Procedure

Participants completed the same demographic questions, personality measures, and economic games as Study 1, which were again programmed using Qualtrics Survey Software and administered through the MTurk requester interface. This time, however, the BFAS was presented before the HEXACO-PI-R and the two were separated by several other questionnaires (e.g., Major Life Goals, Roberts and Robins, 2000). In addition, the games of interest were preceded by a social mindfulness task involving the hypothetical selection of specific objects (Van Doesum et al., 2013) and subjective ratings of the payoff structures of social dilemmas (Halevy et al., 2012), both of which were beyond the scope of the aims of the current research. Neither involved any explicit themes of prosociality and were not expected to produce any carryover effects.

Unlike Study 1, participants’ responses to all games were financially incentivized. This was done by informing participants that their decisions for one of the games (which was pre-selected) would be matched to another participant and used to determine their payment at the end of the session. This approach is similar to the Conditional Information Lottery, which is a standard procedure in the literature (Bardsley, 2000). In the help and hurt reciprocity conditions, participants were asked to indicate their responses using the strategy method and assume that they would be matched to a partner who had picked a given move. Game payoffs were represented by points that corresponded with real dollar amounts at a rate of 1 point to US$0.10. Bonus payments were then provided to participants at the end of the study using their anonymous response identification codes.

Participants completed the same two attention checks as in Study 1. Ten participants (3.8%) were excluded for failing at least one of these checks. The show-up fee was US$8.00, in addition to bonus payments earned from study tasks (US$0.50). The median time spent on the study was 42 min.

### Results and Discussion

#### Preliminary Statistics

##### Game decisions

Mean allocations to a partner are presented in the right panel of **Figure 2.** Comparing across studies, all three dictator allocations were significantly lower in the incentivized Study 2 than the hypothetical Study 1 (*p*s < 0.001). Conversely, all but one generosity allocation (where a partner had previously helped the participant, *p* = 0.16) were significantly higher in Study 2 than Study 1 (*p*s < 0.05).

A 2 (game type: dictator vs. generosity) × 3 (reciprocity: baseline, help, and hurt) repeated measures ANOVA was performed with Greenhouse-Geisser corrections for sphericity violations of reciprocity, χ^2^(2) = 16.00, *p* < 0.001 (𝜀 = 0.94), and its interaction with game type, χ^2^(2) = 10.34, *p* = 0.01 (𝜀 = 0.96). The results in Study 1 were replicated here, including main effects for game type, *F*(1,255) = 253.20, *p* < 0.001, η_p_^2^ = 0.50, and reciprocity, *F*(1.89,480.67) = 9.40, *p* < 0.001, η_p_^2^ = 0.04. There was also an interaction between game type and reciprocity, *F*(1.92,490.44) = 3.87, *p* = 0.02, η_p_^2^ = 0.02, which had been marginally significant (*p* = 0.09) in Study 1. *Post hoc* comparisons with Bonferroni corrections revealed that the effect for reciprocity applied only to dictator games. Dictator allocations were significantly higher in the help games (*M* = 3.74) compared with the baseline (*M* = 3.20, *p* < 0.001), and hurt games (*M* = 3.28, *p* < 0.001), but there were no significant differences across reciprocity conditions for the generosity games (all *p*s > 0.30).

##### Demographic variables

Again, age was not significantly correlated with any game decisions. There was an interaction between gender and game type when gender was included in the 2 (game type) × 3 (reciprocity) repeated measures ANOVA, *F*(1,254) = 15.32, *p* < 0.001, η_p_^2^ = 0.06, shown in the right panel of **Figure 3.** Women allocated significantly more than men in dictator games, *M*s = 3.79 vs. 3.13, *t*(254) = 2.59, *p* = 0.01, but this was reversed in the generosity game, where, as in Study 1, men allocated significantly more than women, *M*s = 7.57 vs. 6.45, *t*(254) = 3.38, *p* = 0.001. All main effects and interactions for game type and reciprocity were replicated when including gender.

##### Prosocial personality traits

Bivariate correlations between prosocial personality traits are shown in **Table 2** and were generally consistent with those in Study 1. However, HEXACO agreeableness was more strongly correlated with all other prosocial traits in Study 2 than in Study 1.

#### Personality Predictors of Game Allocations

##### Bivariate correlations

Bivariate correlations between game allocations and prosocial personality traits are shown in **Table 3** (see Supplementary Tables S1 and S2 in the Supplementary Material for correlations with all personality traits). Compared with Study 1, a stronger pattern of correlations was seen for honesty-humility, where it was again associated with dictator (*r*_s_s = 0.21–0.31)—but not generosity (*r*_s_s = -0.08 to -0.02)—allocations. In contrast to Study 1, however, HEXACO agreeableness was not associated with allocations in any game (*r*_s_s = -0.02–0.07).

##### Repeated measures ANCOVAs

A series of 2 (game type) × 3 (reciprocity) repeated measures ANCOVAs was again performed for each personality model with the relevant traits standardized and entered simultaneously as covariates (see **Tables 4** and **5**; **Figure 4**).

Within the Big Five model, there was again a main effect for agreeableness, *F*(1,254) = 7.12, *p* = 0.01, η_p_^2^ = 0.03. Replacing this with covariates for politeness and compassion revealed a unique main effect for politeness only, *F*(1,253) = 17.89, *p* < 0.001, η_p_^2^ = 0.07, and not compassion, *F*(1,253) = 1.87, *p* = 0.17, η_p_^2^ = 0.01. Unlike Study 1, there was a significant interaction between compassion and game type, *F*(1,253) = 7.70, *p* = 0.01, η_p_^2^ = 0.03. Follow-up analysis revealed that compassion was associated with lower allocations in generosity games, *F*(1,253) = 7.20, *p* = 0.01, η_p_^2^ = 0.03, but did not have a main effect in dictator games when politeness was controlled for, *F*(1,253) = 2.39, *p* = 0.12, η_p_^2^ = 0.01.

Within the HEXACO model, there was a main effect for honesty-humility, *F*(1,253) = 7.02, *p* = 0.01, η_p_^2^ = 0.03, but not agreeableness *F*(1,253) = 0.003, *p* = 0.95, η_p_^2^ < 0.001. Whereas the interaction for HEXACO agreeableness observed in Study 1 fell short of significance here, *F*(1,253) = 2.62, *p* = 0.11, η_p_^2^ = 0.01, there was again a significant interaction between honesty-humility and game type, *F*(1,253) = 11.48, *p* = 0.001, η_p_^2^ = 0.04. As in Study 1, this revealed a significant positive effect of honesty-humility in dictator games, *F*(1,253) = 26.98, *p* < 0.001, η_p_^2^ = 0.10, but not in generosity games, *F*(1,253) = 0.68, *p* = 0.41, η_p_^2^ = 0.003.

The above analyses were repeated and the findings were largely the same when gender was included as an additional term (see Supplementary Tables S3–S5 in the Supplementary Material).

### Summary

The incentivized results of Study 2 replicated many of the main findings from the hypothetical paradigm of Study 1. Again, there was clear evidence of inequality aversion, generosity, and positive reciprocity, which were moderated by gender. When we examined the role of prosocial personality traits, honesty-humility once more interacted with game type, predicting greater allocations in dictator—but not generosity—games. In the Big Five model, we again observed a main effect of politeness—but not compassion—which was globally and uniquely associated with greater allocations across all games.

However, the results of Study 2 also introduced two non-trivial differences compared with Study 1. First, the previous interaction between agreeableness and game type in the HEXACO model disappeared in the incentivized paradigm. In fact, HEXACO agreeableness was not associated with allocations of any kind. Second, a novel and unpredicted interaction with game type emerged for compassion in the Big Five model, in which it was not related to dictator allocations, but predicted lower allocations in the generosity game, once politeness was controlled for. This combination of consistent and less consistent findings across the two studies demonstrates the importance of replication and comparisons across incentivized and hypothetical paradigms.

## General Discussion

Prosociality is a complex, multidimensional construct, yet previous research on personality and social preferences has largely focused on simple games and broad trait domains. Expanding on this literature, we developed a novel behavioral paradigm (inspired by Charness and Rabin, 2002), which integrated multiple social preferences using slight variations of the dictator game. We ran two studies—one with hypothetical decisions and one with incentivized games—across two large, and relatively diverse community samples to identify consistent effects. The findings provide clear evidence of inequality aversion, generosity, and positive reciprocity, which we mapped to a framework of distinct prosocial personality traits. This highlighted the unique roles of politeness from the Big Five model, honesty-humility from the HEXACO model, and more tentatively, traits reflecting irritability, anger, and (a lack of) tolerance and forgiveness.

The sizes for these effects are consistent with those previously observed for the role of personality in economic games, where the sample-size weighted average correlation with dictator allocations was *r*_s_ = 0.20 for Big Five agreeableness (Zhao et al., 2016) and *r*_s_ = 0.25 and *r* = 0.29 for HEXACO honesty-humility (Hilbig et al., 2015a; Zhao et al., 2016). Though they may initially appear modest, these correlations—particularly for HEXACO honesty-humility—are at least as large as the average effect size in social and personality psychology (*r* = 0.21; Richard et al., 2003; Fraley and Marks, 2007), and fall within the middle third of effect sizes in psychology as a whole (Hemphill, 2003). These findings will be discussed in detail in the following sections, with a focus on the robust and replicable effects across both two studies.

### Beyond Egalitarianism: Evidence for Generosity and Positive Reciprocity

In line with a large body of literature, our two studies showed that humans are responsive to additional social preferences that stray from both narrow self-interest and inequality aversion. The findings from the generosity game correspond to previous research showing that many individuals are willing to assist others even when it means being *relatively* less well off, as long as absolute costs are minimal (Charness and Rabin, 2002; Engelmann and Strobel, 2004; Güth et al., 2012). Generosity may be crowded out by the trade-off between self- and other-interests in the dictator game, but when it is costless, “most of us try to make the world a better place” (Güth et al., 2009, p. 13). Likewise, many real-world gestures of prosociality, such as giving directions to a stranger and offering a seat on public transport, are ubiquitous precisely because they are relatively inexpensive forms of benevolence.

In contrast, we found mixed results for reciprocity, with consistent evidence of positive—but not negative—reciprocity across both studies. This supports the idea that negative and positive reciprocity are indeed independent processes and are not driven by the same motivations (Yamagishi et al., 2012; Ackermann et al., 2014). Our results are also reminiscent of the original findings by Charness and Rabin (2002), where there was evidence of positive reciprocity but fewer acts of negative reciprocity, even when it was free to punish a misbehaving partner. This may reflect similar sentiments as those in the generosity game, in that individuals are generally benevolent—or at least non-spiteful—when the stakes are relatively inexpensive. In addition, we found no consistent effects for personality with respect to positive or negative reciprocity, suggesting that individual differences in the propensity to reciprocate are subsumed more generally within broader prosocial tendencies.

Other factors may also contribute to the lack of negative reciprocity in our data. First, all decisions in the games were gain-framed. Even when a partner “hurt” a participant, it simply prevented them from receiving a higher amount rather than incurring a personal loss, which may have been too weak to provoke negative reciprocity. Second, the initial payoff combination (15 for the participant, 10 for the partner) declined by the partner in the hurt conditions of the dictator game was already unequal, which may have convinced participants that their partner’s decision to pass on this offer was justified and not deserving of retaliation. Third, the assessment of different social preferences within a single paradigm may trigger a desire among participants to behave consistently, thus artificially increasing consistency in behavior and nullifying any effects for negative reciprocity. However, the differential patterns of responding across generosity and positive reciprocity conditions provide evidence against any such response set. Future investigations using loss-framed manipulations, different configurations of payoffs, and measurements separated by time may be more appropriate for investigating negative reciprocity.

### Women More Egalitarian, Men More Generous?

One interesting finding to emerge across both studies was the interaction between gender and game type, with men consistently allocating more than women in the (costless) generosity games. In the (costly) dictator game, however, women allocated more than men in incentivized games while there were no gender differences in hypothetical responses. But given that decisions in the latter are already a costless form of prosociality—relying on words rather than actions—the absence of a gender gap here may reflect overestimates of allocations among men relative to women. Hence, while women were more inequality averse, they were not necessarily more altruistic when this involved promoting the welfare of others over and above their own.

Although these results were unpredicted and unrelated to the aims of this research, they provide a clear replication of previous research on gender and social preferences. Several studies have shown that women are more prosocial in simple dictator games, while men are more prosocial when the price of giving drops and when giving or cooperating maximizes efficiency (Eckel and Grossman, 1998; Andreoni and Vesterlund, 2001; Croson and Gneezy, 2009; Kuhn and Villeval, 2015). Even in middle childhood and early adolescence, girls more often than boys select egalitarian allocations of wealth over both selfish and generous allocations (Fehr et al., 2013).

These findings correspond to a wider literature on gender differences in preferences toward social and political inequality (i.e., social dominance orientation), which are largely stable across nations and cultures (Pratto et al., 1994, 1997; Sidanius et al., 2000). Such differences in egalitarianism are believed to arise from evolutionary differences in reproductive strategies, in particular, the accumulation of economic resources and status for male, rather than female, reproductive success (Sidanius et al., 2000). Similarly, the literature on desirable mate qualities and costly signaling indicates that men may engage in greater acts of conspicuous consumption as a display of generosity and resources to increase prestige and status (Griskevicius et al., 2007). In the current paradigm, the safest and easiest way of doing this without putting one’s actual stakes at risk is through costless allocations in the generosity game.

### Politeness as a General Prosocial Tendency in Economic Games

A prominent finding was that the politeness aspect of Big Five agreeableness consistently predicted greater overall allocations in both studies. Although we observed a trend for a main effect of compassion when decisions were hypothetical, this disappeared altogether in the incentivized paradigm. These results are in keeping with previous research demonstrating that politeness—rather than compassion—drives egalitarian allocations in the dictator game, with the divergence between the two clearest in incentivized rather than hypothetical paradigms (Zhao et al., 2016).

This unique effect of politeness suggests that prosociality in these decontextualized and neutrally framed paradigms is a function of the tendency to respect others and to adhere to social norms rather than emotional concern for others’ wellbeing. While compassion plays a fundamental role in real-world forms of prosociality (Eisenberg and Miller, 1987; Bekkers, 2006) and the related construct of empathy is theorized to be the primary conduit through which humans engage in altruistic behavior (Batson, 1991), compassionate motives may not be elicited given the impersonal nature of economic games. This has important implications for the ecological validity of economic games, suggesting that social preferences and behaviors measured in these games only capture a limited form of norm-based prosociality. Indeed, in their commentary more than 20 years ago, Camerer and Thaler (1995) argued that the outcomes of such games reveal more about the economics of manners and etiquette than they do about altruism, which is empirically supported by the current findings.

### HEXACO Honesty-Humility, Agreeableness, and the Limits of Prosociality

A second major finding to appear consistently across studies was the interaction between honesty-humility and game type, where it predicted greater allocations in the dictator game but played no role in the generosity game. Honesty-Humility has been consistently linked to fair and prosocial (or at least the absence of antisocial) behaviors when there are personal profits to be made, such as delinquency (e.g., stealing money; Dunlop et al., 2012), workplace ethics and integrity (Lee et al., 2005; Cohen et al., 2014), and dishonesty (Hilbig and Zettler, 2015). Notably, across both Big Five and HEXACO models, honesty-humility is the trait most strongly and frequently associated with dictator allocations (Zhao and Smillie, 2015). Surprisingly, we found that this link to prosociality disappears when such decisions do not involve a personal cost. In the generosity game where individuals could maximize their partner’s payoffs for free, those high on honesty-humility allocated no differently from their low-scoring counterparts.

On the one hand, this implies that there are limits on the prosociality encompassed by honesty-humility, and, contrary to previous evidence (Hilbig et al., 2015b), suggests that honesty-humility is more closely tied to egalitarianism and fairness than benevolence. On the other hand, further inspection of **Figure 4** shows that this interaction is more strongly driven by those at the *low* pole of honesty-humility. Although they are selfish when they can personally profit, they are neither competitive nor vindictive—and indeed appear concerned about efficiency—once their own stakes are secured.^3^ These results highlight the importance of situational context in the expression of personality traits: Given that HEXACO honesty-humility represents the tendency to cooperate with others despite the opportunity for exploitation (i.e., active cooperation), it is no longer elicited when there is no invitation to exploit in the non-constant-sum structure of the generosity game^4^.

It is noteworthy that these findings for honesty-humility were accompanied by a complementary pattern of results for HEXACO agreeableness in the hypothetical games. While HEXACO agreeableness did not predict dictator allocations, consistent with previous research (Hilbig et al., 2013), it was associated with greater allocations in the generosity game. These findings are in keeping with the core features of HEXACO agreeableness, which capture individual differences in tolerance, lenience, flexibility, and a lack of irritability or anger (Ashton and Lee, 2007; Ashton et al., 2014). All of these tendencies are antithetical to spite and envy, two major motivations for curbing a partner’s allocation in the generosity game. Nevertheless, we interpret this double dissociation with caution as it was not replicated in the incentivized paradigm, where HEXACO agreeableness was unrelated to any form of social preference.

Interestingly, however, we observed a near-identical interaction for the volatility aspect of Big Five neuroticism in the incentivized paradigm, which captures related constructs (i.e., anger and irritability; DeYoung et al., 2007, DeYoung, 2015) and is strongly negatively associated with HEXACO agreeableness (*r*_s_s = -0.60, -0.62 in Studies 1 and 2, respectively). Bivariate correlations and exploratory analyses for volatility (see Supplementary Tables S1 and S6 in the Supplementary Material) showed a similar but inverted pattern to that previously seen for HEXACO agreeableness. Volatility has been linked to psychopathic traits (Jonason et al., 2013), and may provoke envy and resentfulness when individuals face the prospect of disadvantageous inequality in the generosity game.

### Does Incentivization Pay Off?

The dual studies and their near-identical designs provide a useful comparison of trait effects across incentivized and hypothetical designs, which has been a topic of debate among psychologists and economists (Camerer and Hogarth, 1999; Ariely and Norton, 2007). Recent investigations of prosocial traits in the dictator game suggest that while average allocations drop between hypothetical and incentivized designs, the effects of certain traits—politeness and agreeableness from the Big Five model, and HEXACO honesty-humility—tend to be larger in incentivized paradigms (Zhao et al., 2016). Likewise, we also observed considerable discrepancies in average allocations between the two paradigms, with individuals overestimating dictator allocations when providing hypothetical responses. Such “hypothetical biases” are frequently seen in value elicitation methods, in which individuals overstate their willingness to pay for a given good (in this case, equality; Hertwig and Ortmann, 2001; List and Gallet, 2001). Yet, individuals also underestimated how benevolent or efficiency-maximizing they would be in the generosity game. It appears that in the absence of incentivization, all individuals gravitate toward the equality norm, leading to attenuated individual variation and muted trait effects. With incentivization, new trait effects emerged, including interactions for compassion and volatility. These can be understood in relation to a recent meta-analysis on the role of personality traits in cooperative game behaviors, which found moderating effects of incentivisation on Big Five agreeableness and neuroticism (Ferguson et al., 2015). With incentivization, the effect for agreeableness became stronger while the effect for neuroticism went from weakly positive to negative.

## Conclusion

There have been recent calls for an integrated research agenda between personality psychology and economics (Ferguson et al., 2011). In the current research, we mapped two models of personality onto individual differences in social preferences using a parsimonious behavioral paradigm. In the HEXACO model, honesty-humility (but not agreeableness) uniquely predicted egalitarian, but not generous, allocations of wealth. In the Big Five model, the politeness (but not compassion) aspect of agreeableness was uniquely associated with prosocial allocations of wealth more globally. The findings revealed important insights concerning the sources of heterogeneity in social preferences and the mechanisms driving prosocial behavior in economic games. Together, they demonstrate the value of a joint approach that combines theoretical predictions from personality psychology with behavioral paradigms from experimental economics.

## Author Contributions

Conception and design: LS and KZ. Collection, analysis, and interpretation of data: EF, LS, and KZ. Drafting the article: KZ. Revising the article: EF, LS, and KZ.

## Conflict of Interest Statement

The authors declare that the research was conducted in the absence of any commercial or financial relationships that could be construed as a potential conflict of interest.

## Footnotes

1 In addition, an interstitial scale, altruism, represents a blend of HEXACO honesty-humility, agreeableness, and emotionality (e.g., “I have sympathy for people who are less fortunate than I am”; Ashton et al., 2014). Given its extensive overlap with prosocial domains from both personality models (and the focus of the current study on *distinct* prosocial traits) and its relatively lower reliability (Cronbach’s αs = 0.62, 0.71), data for this scale were not included in the main analysis but can be found in the Supplementary Material (see Supplementary Table S2).

2 While our generosity game was inspired by that designed by Güth et al., (2009, 2012) and Güth (2010), the two are not the same as players in the latter decide on the size of the entire pie so that the partner is the residual claimant. In contrast, our participants directly selected the payoffs for their partner, which aided ease of understanding for participants and allowed comparability with dictator games in our analysis.

3 A future extension is to examine these distinct prosocial traits at an even finer level of analysis, as honesty-humility too can be broken down into four facets: sincerity, greed avoidance, fairness, and modesty (Lee and Ashton, 2004). Our own data indicate a main effect for the modesty facet in both incentivized and hypothetical studies, while the interaction between honesty-humility and game type was driven by sincerity in Study 1 and fairness in Study 2.

4 We thank one of our reviewers for this observation.
