# Supplementary material for: Prosocial Personality Traits Differentially Predict Egalitarianism, Generosity, and Reciprocity in Economic Games
Source: Front Psychol. 2016 Aug 9;7:1137. doi: 10.3389/fpsyg.2016.01137 (PMC4977318; doi:10.3389/fpsyg.2016.01137)
Supplement: Supplementary file 1 [file Table_1.DOCX]

Prosocial Personality Traits Differentially Predict Egalitarianism, Generosity, and Reciprocity in Economic Games

**Online supplementary material**

Table S1

## *Correlations between Big Five Personality Traits and Game Allocations*

|  | Study 1: Hypothetical | | | | | |  | Study 2: Incentivized | | | | | |
| --- | --- | --- | --- | --- | --- | --- | --- | --- | --- | --- | --- | --- | --- |
|  | DG | DG_0_ | DG_15_ | GG | GG_0_ | GG_10_ |  | DG | DG_0_ | DG_15_ | GG | GG_0_ | GG_10_ |
| Neuroticism | -.08 | -.02 | .03 | -.10 | -.07 | -.04 |  | .03 | .12^*^ | .16^*^ | -.15^*^ | -.12 | -.06 |
| Withdrawal | -.05 | -.02 | -.003 | -.08 | -.04 | -.02 |  | .04 | .15^*^ | .16^*^ | -.11 | -.08 | -.03 |
| Volatility | -.09 | -.02 | .07 | -.11 | -.10 | -.05 |  | .02 | .08 | .13^*^ | -.17^**^ | -.14^*^ | -.07 |
| Agreeableness | .20^**^ | .17^**^ | .16^**^ | .02 | .05 | .01 |  | .19^**^ | .11 | .18^**^ | -.01 | .0003 | -.07 |
| Politeness | .17^**^ | .16^**^ | .15^**^ | .02 | .07 | -.01 |  | .17^**^ | .12 | .15^*^ | .07 | .07 | .04 |
| Compassion | .19^**^ | .16^**^ | .13^*^ | .05 | .05 | .05 |  | .17^**^ | .07 | .17^**^ | -.09 | -.07 | -.14^*^ |
| Conscientiousness | .11 | .04 | .06 | -.10 | -.10 | -.07 |  | .07 | -.08 | -.04 | -.02 | -.04 | -.04 |
| Industriousness | .08 | .05 | .07 | -.08 | -.09 | -.07 |  | .04 | -.12 | -.09 | .03 | -.004 | -.04 |
| Orderliness | .12^*^ | .01 | .06 | -.07 | -.08 | -.02 |  | .08 | -.02 | .04 | -.10 | -.09 | -.07 |
| Extraversion | .07 | .18^**^ | .01 | -.02 | .02 | -.01 |  | -.01 | -.16^*^ | -.08 | -.05 | -.07 | -.13^*^ |
| Enthusiasm | .04 | .15^**^ | -.004 | .01 | .003 | -.03 |  | .03 | -.07 | .01 | -.08 | -.07 | -.11 |
| Assertiveness | .07 | .16^**^ | .003 | -.03 | .03 | .03 |  | -.06 | -.20^**^ | -.16^*^ | -.01 | -.06 | -.12 |
| Openness/Intellect | .06 | .13^*^ | .03 | .02 | .07 | .02 |  | .10 | -.02 | .01 | .03 | .01 | -.05 |
| Openness | -.01 | .05 | .01 | -.03 | .01 | .03 |  | .12 | .02 | .11 | -.02 | -.04 | -.10 |
| Intellect | .11^*^ | .18^**^ | .04 | .07 | .13^*^ | .005 |  | .01 | -.08 | -.13^*^ | .11 | .08 | .04 |

*Note.* Correlations calculated using Spearman’s rho. Game allocations indicate amount allocated to partner out of 10 units (i.e., dollars or points). Big Five traits and aspects are measured using the Big Five Aspect Scales (BFAS; DeYoung et al., 2007). DG = Baseline dictator game. DG_0_ = Dictator game after partner’s decision cost the participant the 0 unit payoff. DG_15_ = Dictator game after partner’s decision cost the participant the 15 unit payoff. GG = Baseline generosity game. GG_0_ = Generosity game after partner’s decision cost the participant the 0 unit payoff. GG_10_ = Generosity game after partner’s decision cost the participant the 10 unit payoff. *N* = 304 (Study 1), 256 (Study 2).

**p* < .05. ***p* < .01.

Table S2

## *Correlations between HEXACO Personality Traits and Game Allocations*

|  | Study 1: Hypothetical | | | | | |  | Study 2: Incentivized | | | | | |
| --- | --- | --- | --- | --- | --- | --- | --- | --- | --- | --- | --- | --- | --- |
|  | DG | DG_0_ | DG_15_ | GG | GG_0_ | GG_10_ |  | DG | DG_0_ | DG_15_ | GG | GG_0_ | GG_10_ |
| Honesty-Humility | .20^**^ | .12^*^ | .26^**^ | -.06 | -.04 | -.01 |  | .30^**^ | .21^**^ | .31^**^ | -.04 | -.02 | -.08 |
| Agreeableness | .10 | .03 | -.01 | .17^**^ | .18^**^ | .15^**^ |  | .07 | -.02 | .02 | .04 | .004 | -.02 |
| Altruism (interstitial) | .18^**^ | .14^*^ | .07 | .02 | .11 | .09 |  | .20^**^ | .08 | .20^**^ | -.08 | -.06 | -.12 |
| Emotionality | .09 | .12^*^ | .14^*^ | -.08 | -.05 | .02 |  |  |  |  |  |  |  |
| Extraversion | .05 | .13^*^ | -.02 | .03 | .04 | .02 |  |  |  |  |  |  |  |
| Conscientiousness | .13^*^ | .12^*^ | .15^*^ | -.12^*^ | -.07 | -.08 |  |  |  |  |  |  |  |
| Openness to Experience | .02 | .05 | .001 | .02 | .05 | .02 |  |  |  |  |  |  |  |

*Note.* Correlations calculated using Spearman’s rho. Game allocations indicate amount allocated to partner out of 10 units (i.e., dollars or points). HEXACO traits are measured using the HEXACO Personality Inventory—Revised (HEXACO-PI-R; Lee & Ashton, 2004). DG = Baseline dictator game. DG_0_ = Dictator game after partner’s decision cost the participant the 0 unit payoff. DG_15_ = Dictator game after partner’s decision cost the participant the 15 unit payoff. GG = Baseline generosity game. GG_0_ = Generosity game after partner’s decision cost the participant the 0 unit payoff. GG_10_ = Generosity game after partner’s decision cost the participant the 10 unit payoff. *N* = 304 (Study 1), 256 (Study 2).

**p* < .05. ***p* < .01.

Table S3

## *ANCOVA Results for Main Effects of Prosocial Traits When Gender is Included in the Model*

|  | Study 1: Hypothetical | | | |  | Study 2: Incentivized | | | |
| --- | --- | --- | --- | --- | --- | --- | --- | --- | --- |
| Interaction term | df | *F* | *p* | η_p_^2^ |  | df | *F* | *p* | η_p_^2^ |
| *Big Five Model (B5A only)* | | | | | | | | | |
| B5A | 1, 298 | 15.29 | <.001 | .05 |  | 1, 253 | 9.65 | .002 | .04 |
| *Big Five Model* | | | | | | | | | |
| B5Pol | 1, 297 | 5.65 | .02 | .02 |  | 1, 252 | 20.29 | <.001 | .08 |
| B5Comp | 1, 297 | 4.03 | .05 | .01 |  | 1, 252 | 1.21 | .27 | .01 |
| *HEXACO Model* | | | | | | | | | |
| HEXH | 1, 297 | 2.33 | .13 | .01 |  | 1, 252 | 8.90 | .003 | .03 |
| HEXA | 1, 297 | 5.70 | .02 | .02 |  | 1, 252 | 0.06 | .81 | <.001 |

*Note.* B5 = Big Five Model, measured using the Big Five Aspect Scales (BFAS; DeYoung et al., 2007). B5A = B5 Agreeableness. B5Comp = B5 Compassion. B5Pol = B5 Politeness. HEX = HEXACO Model, measured using the HEXACO Personality Inventory—Revised (HEXACO-PI-R; Lee & Ashton, 2004). HEXA = HEX Agreeableness. HEXH = HEXACO Honesty-Humility. *N* = 301 (Study 1), 256 (Study 2).

Table S4

## *ANCOVA Results for Interactions between Prosocial Traits and Game Type When Gender is Included in the Model*

|  | Study 1: Hypothetical | | | |  | Study 2: Incentivized | | | |
| --- | --- | --- | --- | --- | --- | --- | --- | --- | --- |
| Interaction term | *df* | *F* | *p* | η_p_^2^ |  | *df* | *F* | *p* | η_p_^2^ |
| *Big Five Model (B5A only)* | | | | | | | | | |
| Game × B5A | 1, 298 | 0.16 | .69 | .001 |  | 1, 253 | 0.30 | .58 | .001 |
| *Big Five Model* | | | | | | | | | |
| Game × B5Pol | 1, 297 | 0.05 | .83 | <.001 |  | 1, 252 | 3.71 | .06 | .02 |
| Game × B5Comp | 1, 297 | 0.05 | .82 | <.001 |  | 1, 252 | 5.54 | .02 | .02 |
| *HEXACO Model* | | | | | | | | | |
| Game × HEXH | 1, 297 | 8.16 | .01 | .03 |  | 1, 252 | 7.06 | .01 | .03 |
| Game × HEXA | 1, 297 | 6.22 | .01 | .02 |  | 1, 252 | 1.17 | .28 | .01 |

*Note.* B5 = Big Five Model, measured using the Big Five Aspect Scales (BFAS; DeYoung et al., 2007). B5A = B5 Agreeableness. B5Comp = B5 Compassion. B5Pol = B5 Politeness. HEX = HEXACO Model, measured using the HEXACO Personality Inventory—Revised (HEXACO-PI-R; Lee & Ashton, 2004). HEXA = HEX Agreeableness. HEXH = HEXACO Honesty-Humility. *N* = 301 (Study 1), 256 (Study 2).

Table S5

## *ANCOVA Results for Interactions between Prosocial Traits and Reciprocity When Gender is Included in the Model*

|  | Study 1: Hypothetical | | | |  | Study 2: Incentivized | | | |
| --- | --- | --- | --- | --- | --- | --- | --- | --- | --- |
| Interaction term | *df* | *F* | *p* | η_p_^2^ |  | *df* | *F* | *p* | η_p_^2^ |
| *Big Five Model (B5A only)* | | | | | | | | | |
| Reciprocity × B5A | 1.84, 548.96 | 0.71 | .48 | .002 |  | 1.89, 477.11 | 0.27 | .75 | .001 |
| *Big Five Model* | | | | | | | | | |
| Reciprocity × B5Pol | 1.84, 547.26 | 0.73 | .47 | .002 |  | 1.89, 475.26 | 0.22 | .79 | .001 |
| Reciprocity × B5Comp | 1.84, 547.26 | 0.03 | .97 | <.001 |  | 1.89, 475.26 | 0.01 | .99 | <.001 |
| *HEXACO Model* | | | | | | | | | |
| Reciprocity × HEXH | 1.85, 548.31 | 2.57 | .08 | .01 |  | 1.89, 476.27 | 0.18 | .82 | .001 |
| Reciprocity × HEXA | 1.85, 548.31 | 0.39 | .66 | .001 |  | 1.89, 476.27 | 1.75 | .18 | .01 |

*Note.* B5 = Big Five Model, measured using the Big Five Aspect Scales (BFAS; DeYoung et al., 2007). B5A = B5 Agreeableness. B5Comp = B5 Compassion. B5Pol = B5 Politeness. HEX = HEXACO Model, measured using the HEXACO Personality Inventory—Revised (HEXACO-PI-R; Lee & Ashton, 2004). HEXA = HEX Agreeableness. HEXH = HEXACO Honesty-Humility. *N* = 301 (Study 1), 256 (Study 2).

Table S6

## *Results for 2 (Game Type) × 3 (Reciprocity) Repeated Measures ANCOVA with the Volatility Aspect of Big Five Neuroticism as a Covariate*

|  | Study 1: Hypothetical | | | |  | Study 2: Incentivized | | | |
| --- | --- | --- | --- | --- | --- | --- | --- | --- | --- |
| Term | *df* | *F* | *p* | η_p_^2^ |  | *df* | *F* | *p* | η_p_^2^ |
| *Main effects* | | | | | | | | | |
| Game Type | 1, 298 | 226.87 | <.001 | .43 |  | 1, 253 | 248.01 | <.001 | .50 |
| Reciprocity | 1.84, 548.28 | 16.90 | <.001 | .05 |  | 1.90, 479.46 | 9.41 | <.001 | .04 |
| Gender | 1, 298 | 6.18 | .01 | .02 |  | 1, 253 | 1.11 | .29 | .004 |
| *Interactions* |  |  |  |  |  |  |  |  |  |
| Game Type × Reciprocity | 1.91, 569.03 | 2.71 | .07 | .01 |  | 1.92, 486.59 | 3.23 | .04 | .01 |
| Game Type × Volatility | 1, 298 | 0.59 | .44 | .002 |  | 1, 253 | 4.60 | .03 | .02 |
| Game Type × Gender | 1, 298 | 9.88 | .002 | .03 |  | 1, 253 | 12.58 | <.001 | .05 |
| Reciprocity × Volatility | 1.84, 548.28 | 1.22 | .29 | .004 |  | 1.90, 479.46 | 5.90 | .003 | .02 |
| Reciprocity × Gender | 1.84, 548.28 | 5.07 | .01 | .02 |  | 1.90, 479.46 | 0.04 | .95 | <.001 |
| *Covariates* |  |  |  |  |  |  |  |  |  |
| Volatility | 1, 298 | 2.15 | .14 | .01 |  | 1, 253 | 0.99 | .32 | .004 |

*Note.* Volatility measured using the Big Five Aspect Scales (BFAS; DeYoung et al., 2007). *N* = 301 (Study 1), 256 (Study 2).
